# Diametrically opposed effects of hypoxia and oxidative stress on two viral transactivators

**DOI:** 10.1186/1743-422X-7-93

**Published:** 2010-05-10

**Authors:** Amber T Washington, Gyanendra Singh, Ashok Aiyar

**Affiliations:** 1 Department of Microbiology, Immunology and Parasitology, LSU Health Sciences Center, 1901 Perdido Street, New Orleans, LA 70112, USA; 2 Stanley S. Scott Cancer Center, 533 Bolivar Street, LSU Health Sciences Center, New Orleans, LA 70112, USA

## Abstract

**Background:**

Many pathogens exist in multiple physiological niches within the host. Differences between aerobic and anaerobic conditions are known to alter the expression of bacterial virulence factors, typically through the conditional activity of transactivators that modulate their expression. More recently, changes in physiological niches have been shown to affect the expression of viral genes. For many viruses, differences in oxygen tension between hypoxia and normoxia alter gene expression or function. Oxygen tension also affects many mammalian transactivators including AP-1, NFkB, and p53 by affecting the reduced state of critical cysteines in these proteins. We have recently determined that an essential cys-x-x-cys motif in the EBNA1 transactivator of Epstein-Barr virus is redox-regulated, such that transactivation is favoured under reducing conditions. The crucial Tat transactivator of human immunodeficiency virus (HIV) has an essential cysteine-rich region, and is also regulated by redox. Contrary to EBNA1, it is reported that Tat's activity is increased by oxidative stress. Here we have compared the effects of hypoxia, oxidative stress, and cellular redox modulators on EBNA1 and Tat.

**Results:**

Our results indicate that unlike EBNA1, Tat is less active during hypoxia. Agents that generate hydroxyl and superoxide radicals reduce EBNA1's activity but increase transactivation by Tat. The cellular redox modulator, APE1/Ref-1, increases EBNA1's activity, without any effect on Tat. Conversely, thioredoxin reductase 1 (TRR1) reduces Tat's function without any effect on EBNA1.

**Conclusions:**

We conclude that oxygen partial pressure and oxidative stress affects the functions of EBNA1 and Tat in a dramatically opposed fashion. Tat is more active during oxidative stress, whereas EBNA1's activity is compromised under these conditions. The two proteins respond to differing cellular redox modulators, suggesting that the oxidized cysteine adduct is a disulfide bond(s) in Tat, but sulfenic acid in EBNA1. The effect of oxygen partial pressure on transactivator function suggests that changes in redox may underlie differences in virus-infected cells dependent upon the physiological niches they traffic to.

## Background

The human body contains multiple niches that vary greatly in oxygen tension. For example, lymph nodes have oxygen partial pressure (pO_2_) ranging from 10-20 Torr (1-2.5% O_2_) [1-3]. In contrast, peripheral blood has an average level of 10-12% oxygen [*ibid*, [4]]. It is known that the activity of many mammalian transactivators is sensitive to changes in oxygen tension, leading to niche-specific gene expression patterns [5-9]. For years it has been noted that oxidative conditions alter gene expression in many pathogens [10-15]. Furthermore, oxygen tension is known to affect the activity of many viral proteins, including transactivators, thus changing the outcome of viral infection [16-18].

One such virus that displays this characteristic is the lymphotropic human herpesvirus, Epstein-Barr virus (EBV). EBV is latent in B-cells that exist in the peripheral circulation as non-dividing memory B-cells; within lymph nodes EBV-infected cells become proliferating blasts that secrete antibody [19,20]. These two dramatically distinct cellular phenotypes result from two different viral gene expression patterns during latency [*ibid*]. Recent results indicate that the EBV transactivator, Epstein-Barr nuclear antigen 1 (EBNA1), is regulated by oxygen tension [18]. Under hypoxic or reducing conditions, EBNA1 is active as a transactivator and drives viral gene expression required for cell proliferation. For EBNA1, the redox state of a pair of cysteines in a conserved cys-x-x-cys motif governs its ability to transactivate [*ibid*].

Similar to EBNA1, the HIV-1 Tat protein contains a redox-sensitive cysteine-rich region with multiple cys-x-x-cys motifs that is essential for Tat's ability to transactivate [21-24]. Although it was initially believed that Tat's cysteine-rich region was used to coordinate zinc [25,26], it is now known that intramolecular disulfide bonds between the cysteine sulfhydryl groups are essential for transactivation, whereas zinc coordination is not [27-29]. Reiterating the importance of these disulfide bonds, recent reports indicate that oxidative conditions increase Tat's capacity to transactivate [24], whereas hypoxia reduces transactivation [30].

Currently, there are two known mechanisms by which oxygen tension is sensed by cysteine. High intracellular oxygen tension results in disulfide bond formation between neighbouring cysteine sulfhydryl groups. Alternatively, sulfhydryl groups can be oxidized to sulfenic acid. While both changes can be reversed under conditions of low oxygen tension, agents that reduce disulfide bonds cannot reduce sulfenic acid to sulfhydryl [31].

In this report, we have examined the effects of oxygen tension and oxidative stress on EBNA1 and Tat. Our results indicate that changes in redox have opposing effects on these two viral transactivators: EBNA1 is more active under reducing conditions, whereas Tat is more active under oxidative conditions. There is also a dichotomy in the cellular redox modulators that affect the function of EBNA1 and Tat. A redox modulator that reduces sulfenic acid to sulfhydryl increases EBNA1's activity, but has no effect on Tat. Conversely, modulators that reduce disulfide bonds decrease transactivation by Tat, but have no effect on EBNA1. We discuss the significance of our findings in the context of EBNA1's and Tat's roles during EBV and HIV associated pathogenesis.

## Methods

### Effector Plasmids

AGP441, used to express a C-terminally 3xFLAG epitope EBNA1, was made by adding a 3xFLAG epitope tag to the C-terminus of EBNA1 in plasmid 1553 [32]. The EBNA1-derivative used here contains an internal deletion in the gly-gly-ala repeat but transactivates as well as wild-type [32] AGP535, used to express a C-terminally 3xFLAG tagged HIV-1 Tat, was constructed by replacing the EBNA1 ORF in AGP441 with the Tat sequence from the prototypic HXB2 clone of HIV-1. In AGP441 and AGP535, epitope tagged EBNA1 and Tat are expressed from the CMV immediate early promoter. pcDNA3.1, the empty parent expression plasmid, was used for control transfections. AGP494 and AGP559 were used to express APE1/Ref-1 and thioredoxin reductase-1. Plasmid 2145, which expresses EGFP under the control of the CMV immediate early promoter, was used to correct for transfection efficiency [33].

### Reporter Plasmids

The EBNA1 reporter plasmid, AGP95, has been described previously [33]. It contains 20 EBNA1-binding sites, termed the family of repeats (FR), placed 5' to a minimal HSV-1 TK promoter (TKp) [34] luciferase reporter cassette. AGP546, the Tat reporter plasmid was constructed by excising FR from AGP95, and then inserting the TAR element from HIV-1 (LAV) between TKp and the luciferase gene. Similar to Tat-responsive, TAR-containing reporters described before [35], in AGP546 the first nucleotide transcribed is the first nucleotide of U5. Plasmid AGP47, TKp-luciferase, was used in some experiments as a control plasmid. This plasmid lacks EBNA1 binding sites, and there is no TAR element in the luciferase transcript from TKp.

### Cell Culture and Transfections

The human cell epithelial cell-line, C33a, was propagated in DMEM:F12 (1:1) supplemented with 5% bovine calf serum. Cells were maintained in a 5% CO_2 _incubator under normoxic (20% O_2_), or hypoxic (4% O_2_) conditions. Cells were transfected as described previously. Pharmacologic agents including menadione, paraquot dichloride, sodium selenite, beta-mercaptoethanol, glutathione, and N,N,N',N'-tetrakis (2 pyridylmethyl) ethylenediamine (TPEN) were purchased from Sigma (St. Louis, MO), and added 6 hours post-transfection, and cells were harvested 18-20 hours post-addition. Control cells were treated to the vehicle for the specific pharmacologic agent being tested. Transfections were normalized using the GFP expression plasmid, 2145 by FACS profiling a fraction of each transfection to determine the fraction of live-transfected cells (GFP-positive cells that did not stain with propidium iodide). This analysis was used to correct for differences in transfection efficiency or cell survival post-transfection as described previously [18,33,36,37].

### Hypoxia Conditions

Cells used in hypoxia experiments were grown in a sealed modular incubation chamber (Billups-Rothenberg, Inc, Del Mar, CA) placed at 37°C. The chamber was flushed with 4% O_2 _(AirGas, Theodore, AL) for five minutes prior to sealing. Chambers were re-equilibrated every 12 hours. When necessary, media changes were performed using media previously equilibrated in a 4% O_2 _atmosphere.

### Luciferase Reporter Assays

For Tat assays, 0.3 μg of the reporter AGP546 (TKp-TAR-luciferase) was co-transfected with 10 μg of the Tat-expression plasmid AGP535, and 0.5 μg of the CMV-GFP plasmid. For EBNA1 assays, 0.3 μg of the reporter AGP95 (FR-TKp-luciferase) was co-transfected with 2 μg of the EBNA1 expression plasmid, AGP441, and the CMV-GFP plasmid as described above. Plasmid AGP47, TKp-luciferase, was used in some experiments as a control plasmid. Cells were harvested 24 hours post-transfection, and analyzed to determine the percent of live-transfected cells, prior to luciferase assays performed as described previously [18,33,36,37].

### Indirect Immunofluorescence Microscopy and Image Deconvolution

Cells transfected with the TAT-3xFLAG or EBNA1-3xFLAG expression plasmids were plated on Type 1 cover slips and processed for immunofluorescence as described previously [18,36,37]. The M2 anti-FLAG mouse monoclonal Ab (Sigma) was used as the primary antibody, and AlexaFluor 488 tagged anti-mouse Ab was used as the secondary Ab. Hoechst 33342 was used as the counter-stain to visualize nuclei. Images were obtained using an inverted Zeiss AxioVision AX10 microscope at 63X using an AxioCam MRm camera. Z-stacks containing fifteen 200 nm optical sections were deconvolved using a constrained iterative Fourier transform.

### Immunoblotting

Immunoblots were performed as described previously using the M2 anti-FLAG mouse mAb (1:1000 dilution) as the primary antibody [38], and horseradish peroxidase conjugated rabbit anti-mouse secondary antibody. Anti-actin primary Abs, ab8226 (Abcam) or A8592 (Sigma) were used to detect beta-actin for as a loading control. Blots were visualized by chemiluminescence as described previously [36-38].

## Results

### Choice of reporter cell-line, and construction of a Tat reporter plasmid

Our experiments comparing the effects of redox on EBNA1 and Tat were performed in C33a cells for the following reasons. Multiple studies indicate that EBNA1 efficiently transactivates an FR-dependent reporter in C33a cells [18,33,37,39]. In addition, we have characterized metal ion requirements and some effects of oxidative stress on EBNA1's ability to transactivate in these cells [18]. Tat is known to transactivate an HIV-LTR luciferase reporter in multiple cell-lines including epithelial lines such as 293 and the Hela derivative TZM-bl. Therefore, after confirming that Tat transactivated an HIV-LTR reporter in C33a cells (data not shown), we chose C33a cells for this study. Studying both transactivators in the same cell-line has permitted comparing them without the interpretational complications caused by using two different cell-lines.

Both reporter plasmids used the minimal TK promoter (TKp), rather than native viral promoters because viral promoters that respond to EBNA1 or Tat contain binding sites for cellular redox-responsive transcription factors [5,7,8,40,41]. Previous studies [18], as well as results reported here, indicate that basal transcription from TKp is not redox-sensitive. For EBNA1, we have used the reporter FR-TKp-luciferase, in which a cluster of 20 EBNA1 binding sites from the EBV genome is placed 5' to a TKp-luciferase reporter cassette [33,39]. We constructed an analogous reporter for Tat by inserting the HIV-1 TAR RNA element between TKp and the luciferase gene. This reporter, TKp-TAR-luciferase, contains 77 nucleotides of HIV-1 sequence from the LAV strain of HIV-1 between the TKp and luciferase [35]. The first nucleotide transcribed in TKp-TAR-luciferase is predicted to be the first nucleotide in the HIV-1 RNA genome.

**Figure 1:**
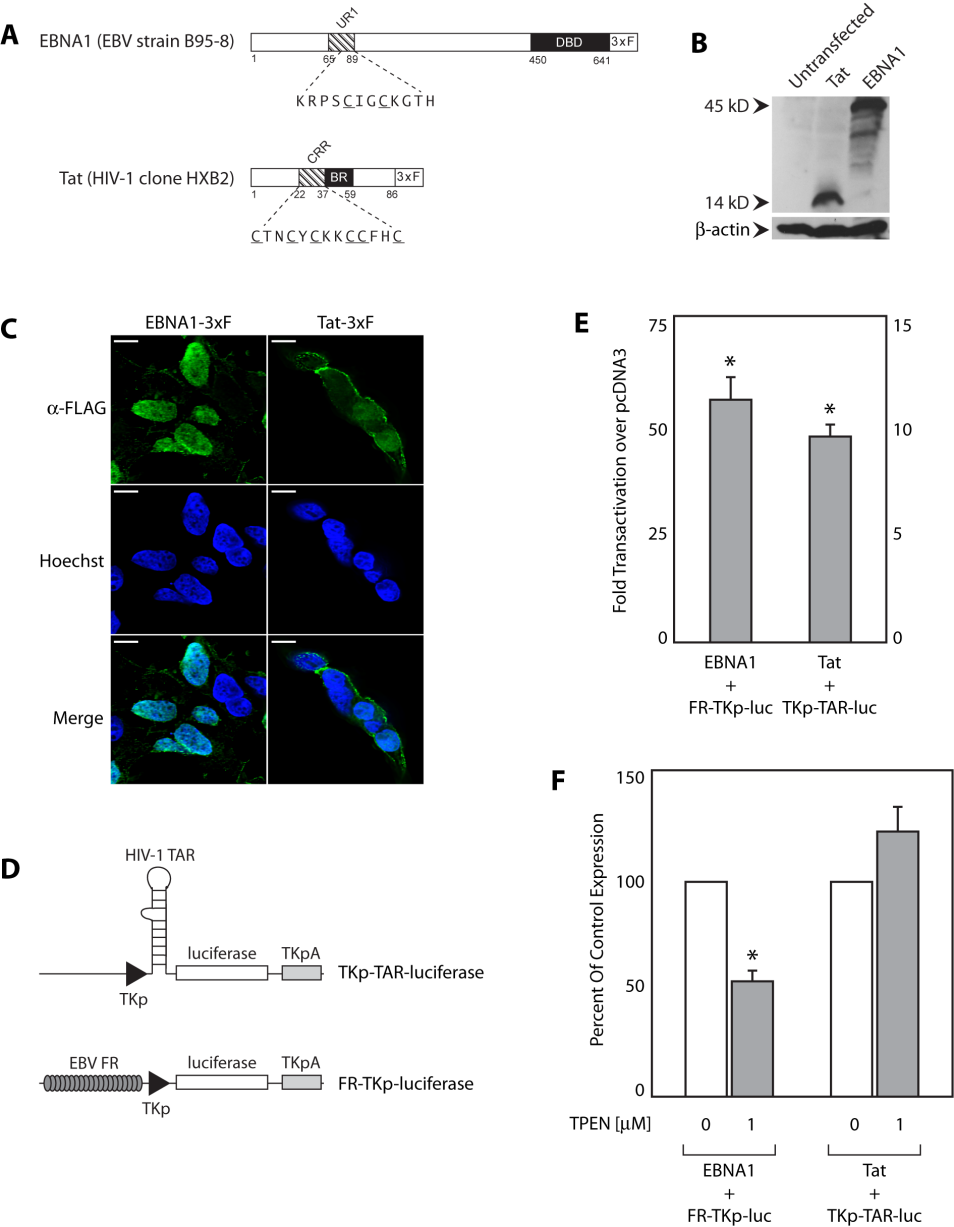
**Characterization of epitope-tagged EBNA1 and Tat**. (A) Diagrams of epitope-tagged EBNA1 and Tat. EBNA1 is 641 a.a. long and binds 20 sites in the EBV FR through its DNA binding domain (DBD). EBNA1's UR1 domain, essential for transactivation, contains a redox-regulated cys-x-x-cys motif. Tat is 87 a.a. long and binds HIV-1 TAR RNA through its basic region (BR) Tat's redox-regulated cysteine-rich (CRR) is required for transactivation. (B) Epitope-tagged EBNA1 and Tat, expressed in C33a cells, were visualized as described in the materials and methods. (C) Indirect immunofluorescence indicates EBNA1 is primarily nuclear, while Tat is nuclear and cytoplasmic. Proteins were visualized as described in the materials and methods. Bars indicate a scale of 10 μM. (D) Diagram of the transcription reporter plasmids. The minimal TK promoter (TKp) in both reporters has -1 to -80 of the HSV-1 TK promoter. The Tat reporter, TKp-TAR-luciferase, contains the HIV-1 TAR between the promoter and the luciferase gene. The EBNA1 reporter, FR-TKp-luciferase, contains the EBV FR 5' to the TKp. The HSV-1 TK polyadenylation signal (TKpA) was used for polyadenylation. (E) 24 hours post-transfection, epitope-tagged EBNA1 transactivates FR-TKp-luciferase 55-fold over the control (pcDNA3) (left-hand scale). Epitope-tagged Tat transactivates TKp-TAR-luciferase 10-fold over pcDNA3 (right-hand scale). (F) Exposure to 1 μM TPEN, a zinc chelator, reduced transactivation of FR-TKp-luciferase by EBNA1 to 50% of control, as observed for native EBNA1. TPEN did not alter transactivation by Tat. The asterisk indicates statistical significance by the Wilcoxon rank-sum test (p < 0.05) over control conditions.

Schematic representations of epitope-tagged EBNA1 and Tat are shown in Figure 1A, emphasizing the domains of these two proteins that are required to bind their cognate recognition sites on DNA or RNA, and the domains that are redox-responsive. EBNA1's DNA-binding domain (DBD) (a.a. 451-641) is used to bind the 20 EBNA1-binding sites in FR [39]. The UR1 domain of EBNA1 (a.a. 65-89) contains a redox-regulated cys-x-x-cys motif that is essential for transactivation [18]. Tat uses its basic region (BR) (a.a. 38-59) to bind TAR, and contains a redox-regulated cysteine-rich region (CRR) (a.a. 22-37) essential for transactivation [23,28]. The expression of these epitope tagged proteins is shown in Figure 1B; neither EBNA1 nor Tat was observed to be extensively degraded within the time-course of these experiments. Indirect immunofluorescence indicated that epitope-tagged EBNA1 and Tat had sub-cellular localizations similar to untagged EBNA1 and Tat (Figure 1C). Tat was observed to be both nuclear and cytoplasmic, whereas EBNA1 was predominantly nuclear. The epitope-tagged versions of EBNA1 and Tat are referred to as EBNA1 and Tat in this report.

The reporter plasmids used to assay transactivation by EBNA1 and Tat are schematically depicted in Figure 1D. As described earlier, both plasmids contain a TKp-luciferase reporter cassette in either an EBNA1 (AGP95) or Tat (AGP546) responsive context. EBNA1 transactivated FR-TKp-luciferase approximately 55-fold over pcDNA3, used as a control effector plasmid, and Tat transactivated TKp-TAR-luciferase approximately 9-fold over pcDNA3 (Figure 1E). Both EBNA1 and Tat can coordinate zinc. However, while EBNA1 needs zinc coordination to transactivate [18], Tat does not [28]. To confirm that the metal-ion (zinc) requirements of the epitope-tagged proteins were unchanged, transfected cells were exposed to TPEN, a chelator with high specificity for Zn^2+ ^and Fe^2+^. TPEN treatment began six hours post-transfection and continued for an additional 18 hours prior to analysis. Treatment with 1 μM TPEN reduced EBNA1's transactivation of FR-TKp-luciferase to 50% of control conditions (Figure 1F), but had no statistically significant effect on transactivation of TKp-TAR-luciferase by Tat, reproducing prior observations made with the native proteins [18,28]. This experiment also confirms that TPEN does not have a non-specific effect on transcription, nor does it directly affect the basal transcription machinery active at the minimal TK promoter (Additional File 1A).

### Hypoxia alters transactivation by Tat and EBNA1

**Figure 2:**
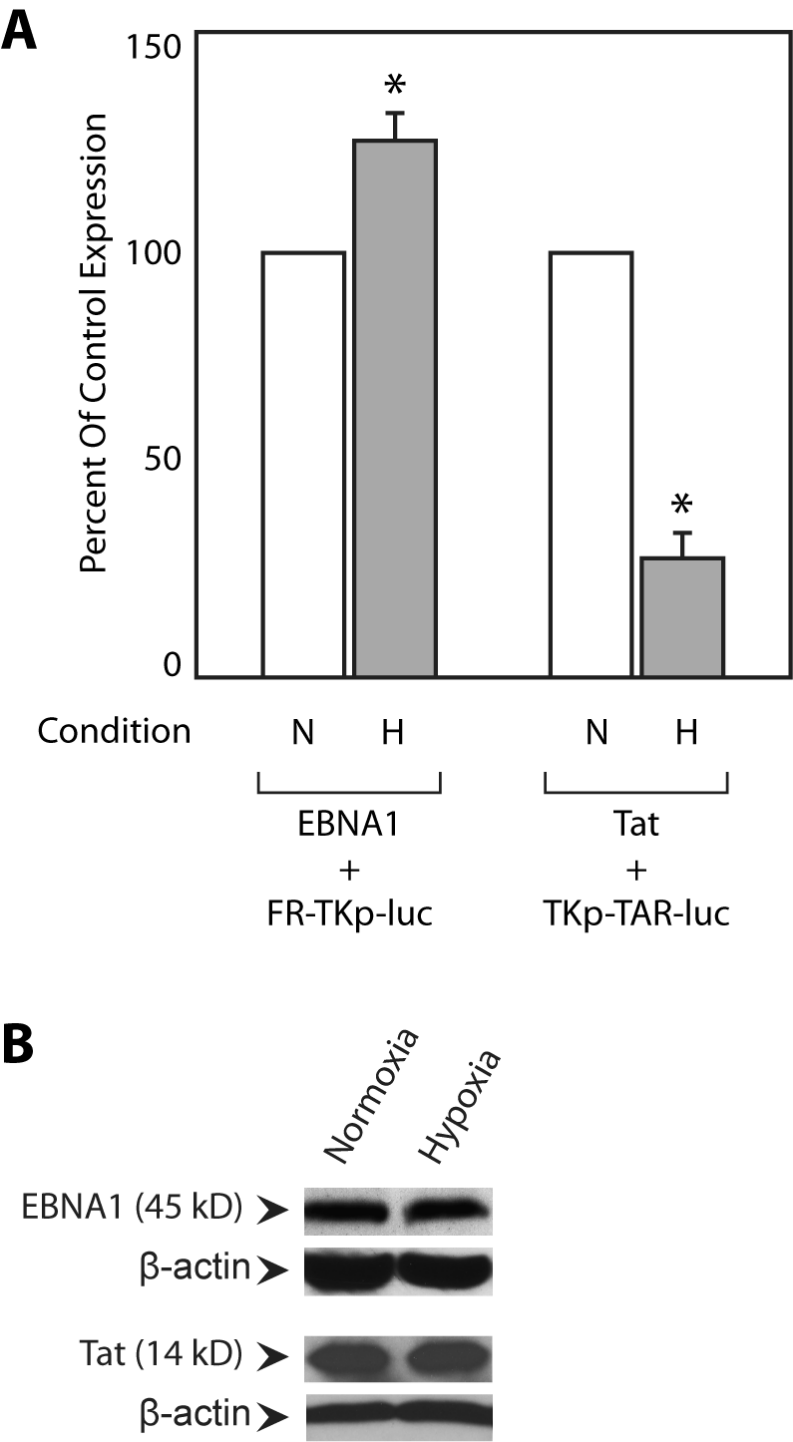
**Hypoxia increases transactivation by EBNA1 and reduces transactivation by Tat**. (A) Transfected C33a cells were split 6 hours post-transfection into aliquots incubated under normoxia (N) or hypoxia (H). Luciferase activity was assayed at 24 hours post-transfection. Transactivation is expressed as a percent of transactivation observed under normoxic (control) conditions. Hypoxia increased transactivation by EBNA1 increased to 125% of normoxic conditions, but decreased transactivation by Tat to 25% of normoxic conditions. (B) Immunoblots indicate that hypoxia did not alter expression of Tat or EBNA1. β-actin was used as a loading control. Asterisks indicate statistical significance by the Wilcoxon rank-sum test (p < 0.05) when comparing results obtained under hypoxia against normoxia.

EBNA1 and Tat contain redox-sensitive cysteines that are essential for transactivation [18,28], and oxidative stress is known to alter the ability of these proteins to transactivate [18,24]. Oxidation modifies cysteines in two distinct ways: 1) by oxidizing adjacent sulfhydryl groups to form inter- or intra-molecular disulfide bods, and 2) by oxidizing cysteines to sulfenic acid and further oxidized derivatives [31]. Hypoxic conditions decrease the generation of intracellular reactive oxygen species and therefore favour the presence of sulfhydryl groups over oxidized derivatives [42]. Therefore, we examined if hypoxia (4% O_2_) altered transactivation by EBNA1 or Tat, shown in Figure 2. Consistent with previous reports (Figure 2A), for EBNA1, hypoxia significantly increased transactivation to 130% over normoxia transactivation, defined as control conditions, within 24 hours of exposure to 4% O_2_. In contrast, 4% O_2 _significantly reduced Tat's capacity to transactivate to 25% of normoxic conditions (Figure 2A), consistent with recently published reports indicating that hypoxia reduces Tat's ability to transactivate whereas depletion of cellular redox modulators increases transactivation [24,30]. The changes in transactivation induced by hypoxic conditions did not result from an altered expression of EBNA1 or Tat during hypoxia (Figure 2B). In addition, this experiment indicates that the augmentative effect of hypoxia on EBNA1 does not result from direct changes to the basal transcription machinery functional at the minimal HSV-1 TK promoter. To confirm that hypoxia does not directly affect the basal transcription machinery active at the TK promoter, expression from reporter AGP47 (TKp-luciferase) was examined under hypoxia and normoxia. No significant difference in reporter expression was observed confirming that hypoxia does not affect basal transcription from TKp (Additional File 1B).

Next, we tested whether agents that increase intracellular oxidative stress altered transactivation by EBNA1 and Tat in a manner opposite to the effect of hypoxia.

### Differing effects of the oxidizing agents menadione and paraquot on Tat and EBNA1

EBV and HIV-1 infected cells reside in anatomical niches that differ in oxygen partial pressure (pO_2_). EBV-infected cells proliferate in niches with low pO_2 _(≤ 4% O_2_) [19,43,44], indicating high levels of viral gene expression in such niches. On the other hand, it is reported that HIV-1 RNA levels are generally lower in anoxic niches such as the brain or CSF, when compared to plasma viral load from the same patient [45,46]. Conversely in peripheral circulation (≥ 10% O_2_) [43,44], EBV-infected cells reside as quiescent memory B-cells, whereas higher levels of HIV RNA is detected in plasma [45,46].

pO_2_-dependent intracellular Fenton reactions generate hydroxyl and superoxide radicals and thereby create a continuous flux of intracellular oxidative stress in response to the extracellular pO_2 _[42]. Normoxia (21% O_2_) increases the rate of radical generation over the hypoxic conditions that are present in most tissues. Cells that are explanted compensate for the increased oxidative stress by over-expressing proteins that scavenge radicals or reduce oxidized adducts [47,48]. It is believed cell-lines that cell-lines are more resistant to pO_2-_induced oxidative stress than primary cells for the same reason [*ibid*]. Therefore, low levels of chemical oxidants can be used under normoxia to increase radical generation and thereby circumvent the difficulty in inducing oxidative stress by solely increasing pO_2 _[49]. Menadione and paraquot are most frequently used to increase intracellular hydroxyl and superoxide radicals [50-52], and were therefore selected as the most suitable oxidizing agents for this study.

**Figure 3:**
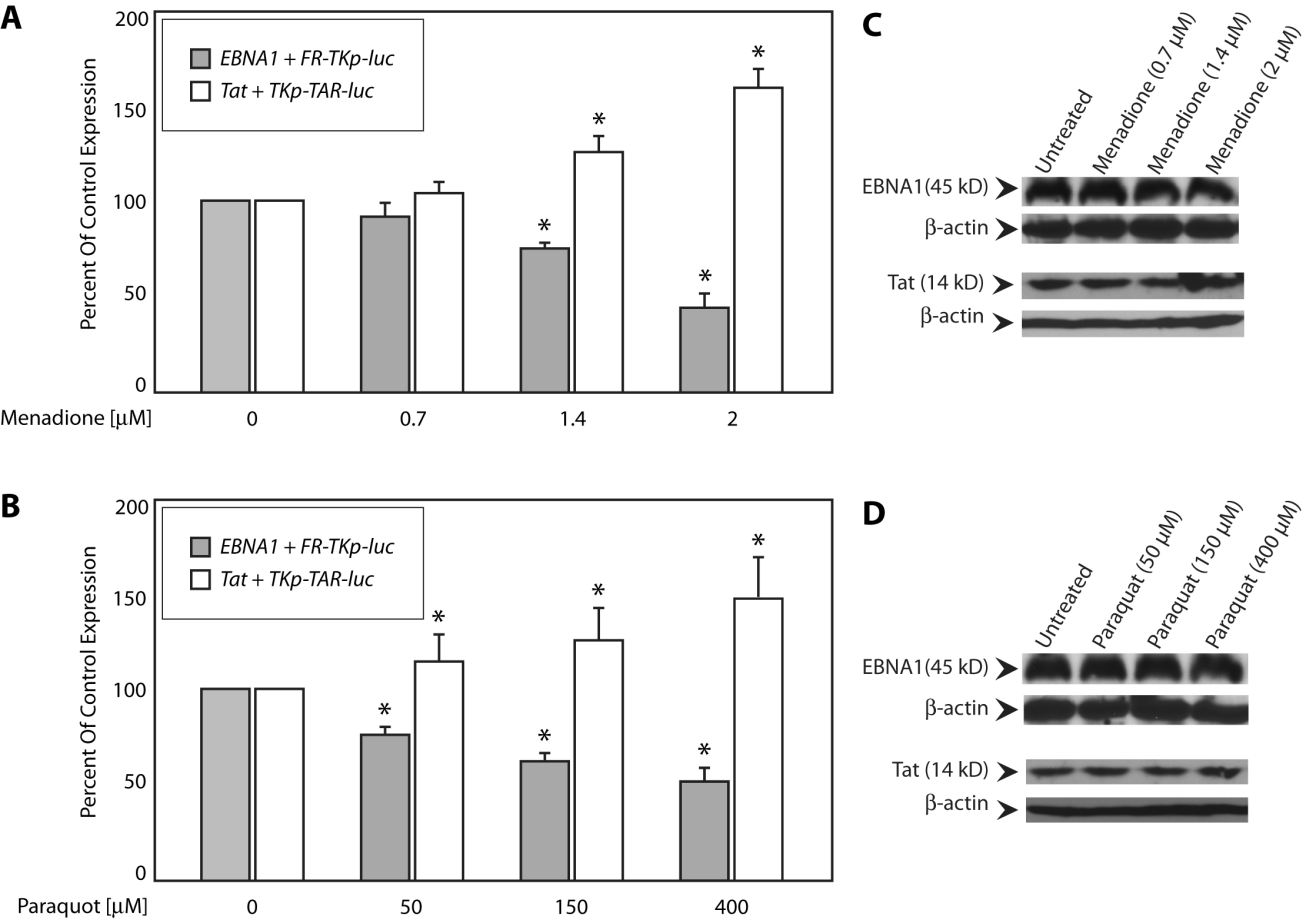
**Oxidative stress induced by menadione and paraquot decrease transactivation by EBNA1, but increase transactivation by Tat**. (A) Transfected C33a cells were split 6 hours post-transfection into aliquots and exposed to the indicated concentrations of menadione, or paraquot (B) for an additional 18 hours, prior to reporter analysis. The inset legend indicates the columns corresponding to each effector/reporter combination. Control cells were vehicle treated. Transactivation is expressed as a percent of transactivation observed in the control cells. Immunoblots indicate that neither menadione (C), nor paraquot (D) altered the expression of EBNA1 or Tat. β-actin was used as a loading control. Asterisks indicate statistical significance by the Wilcoxon rank-sum test (p < 0.05) for treated samples compared to vehicle-treated controls.

For the experiments shown in Figure 3, C33a cells transfected with effector and reporter plasmids were split six hours post-transfection into aliquots that were exposed to the indicated ranges of menadione (Figure 3A) and paraquot (Figure 3B) for 18 hours. At this time, reporter expression was assayed and is indicated as percent of reporter expression observed in the absence of menadione or paraquot (control conditions). As observed previously [18], menadione (Figure 3A) decreased transactivation by EBNA1 in a dose-dependent manner with significant decreases at concentrations at or greater than 1.4 μM. EBNA1's capacity to transactivate the FR-TKp-luciferase reporter was reduced to 50% by 2 μM menadione. In striking contrast, menadione caused a dose-dependent increase in transactivation of TKp-TAR-luciferase by Tat, with significant increases at 1.4 μM menadione and higher. At a concentration of 2 μM menadione, Tat-dependent reporter expression increased to 175% of control. Similar to menadione, paraquot treatment (Figure 3B) reduced transactivation by EBNA1 while increasing transactivation by Tat. For example, 400 μM paraquat increased Tat's activity to 150% of control, but reduced EBNA1's activity to 50% of control (Figure 3B). Changes in transactivation caused by menadione and paraquot did not result from altered expression of EBNA1 or Tat. (Figure 3C, 3D). Oxidative stress also did not affect basal transcription from the TKp (Additional Figure 1C).

### Beta-mercaptoethanol selectively diminishes transactivation by Tat

Oxidation of sulhydryls (-SH) results in either disulfide bond formation (-S-S-) or the progressive formation of sulfenic (-SO), sulfinic (-SO_2_), and sulfonic acid (-SO_3_) [31]. Chemical reductants such as beta-mercaptoethanol or dithiothreitol can reduce disulfide bonds, but have no effect on the other oxidized derivatives of sulhydryl. Therefore, they can be used to distinguish between the two types of adducts that can result from oxidative stress.

**Figure 4:**
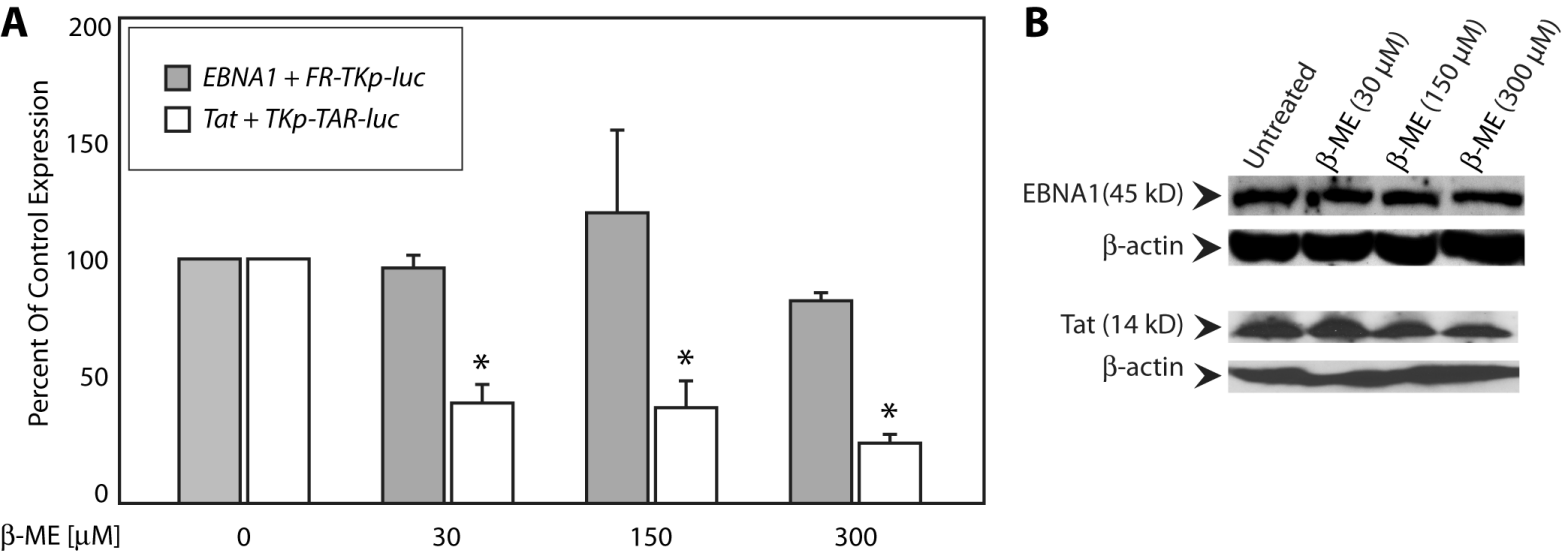
**Beta-mercaptoethanol reduces transactivation by Tat, but has no effect on transactivation by EBNA1**. (A) Transfected C33a cells were split 6 hours post-transfection into aliquots and exposed to the indicated concentrations of beta-mercaptoethanol (β-ME) for an additional 18 hours, prior to reporter analysis. The inset legend indicates the columns corresponding to each effector/reporter combination. Control cells were vehicle treated. Transactivation is expressed as a percent of transactivation in the absence of beta-mercaptoethanol (control conditions). (B) Immunoblots indicate that beta-mercaptoethanol did not affect expression of EBNA1 or Tat. β-actin was used as a loading control. Asterisks indicate statistical significance by the Wilcoxon rank-sum test (p < 0.05) for treated samples compared to vehicle-treated controls.

To evaluate the effect of reducing agents, cells transfected with effector and reporter plasmids were split six hours post-transfection, and aliquots were exposed to a titration of beta-mercaptoethanol (Figure 4A) and dithiothreitol. When assayed 18 hours later concentrations of beta-mercaptoethanol of 30 μM and higher significantly diminished Tat's capacity to transactivate, but had no significant effect on EBNA1. No effect on either protein was observed at 10 μM, and a variable effect on Tat was observed at 20 μM. Between 30-300 μM, beta-mercaptoethanol had no effect on transcription from the minimal TK promoter (Additional File 1D). Deleterious effects on cells were observed at concentrations greater than 300 μM (data not shown).

At 300 μM and less, beta-mercaptoethanol had no effect on cell proliferation or viability. In addition, no effect on the expression of Tat or EBNA1 was observed (Figure 4B). Attempts to confirm these results using dithiothreitol were thwarted by its toxicity on cells. In a single experiment, glutathione at a concentration of 8 μM, reduced transactivation by Tat to 40% of control, without affecting transactivation by EBNA1 (data not shown.

We further dissected these results by examining the effect of over-expressing two common cellular redox modulators, namely AP-endonuclease 1 (APE1/Ref-1) and thioredoxin reductase 1 (TRR1).

### Over-expression of APE1/Ref-1 selectively augments transactivation by EBNA1

**Figure 5:**
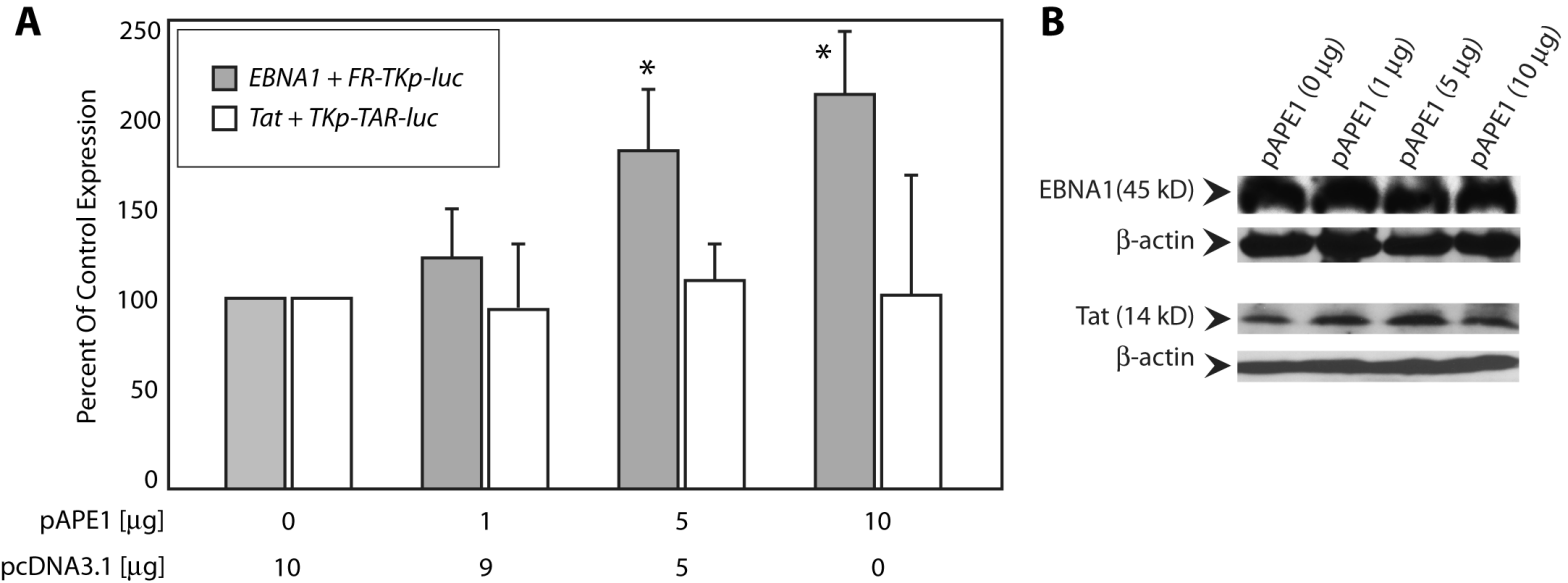
**APE1/Ref-1 increases transactivation by EBNA1, but does not alter transactivation by Tat**. (A) C33a cells were co-transfected with reporter and effector plasmids, and the indicated amount of an APE1/Ref-1 expression plasmid. The backbone expression plasmid, pcDNA3.1 was used to normalize the amount of DNA used in each transfection. Reporter activity was measured 24 hours post-transfection. Transactivation is expressed as a percent of transactivation observed in the absence of co-transfected pAPE1 (control conditions). The inset legend indicates the columns corresponding to each effector/reporter combination. (B) Immunoblots indicate that expression of APE1/Ref-1 did not alter expression of EBNA1 or Tat. β-actin was used as a loading control. Asterisks indicate statistical significance by the Wilcoxon rank-sum test (p < 0.05) for APE1/Ref-1 transfected cells compared to control cells where pcDNA3.1 was co-transfected with the effector and reporter plasmids.

The DNA repair enzyme APE1 (also known as Ref-1) has two functions. It cleaves DNA at apurinic/apyrimidinic sites, and regulates the function of multiple transactivators whose activities are redox-dependent [5-9]. APE1/Ref-1 reduces sulfenic acid back to sulfhydryl [31], although it is unknown whether it can also reduce a disulfide bond. C33a cells were co-transfected with reporter and effector plasmids and variable amounts of an APE1/Ref-1 expression plasmid. Reporter activity was assayed 24 hours post-transfection. As shown in Figure 5A, APE1/Ref-1 significantly augments EBNA1's ability to transactivate to as much as ~200% of control. Transactivation was augmented as a function of increasing the levels of a co-transfected APE1/Ref-1 expression plasmid. This observation, made with epitope-tagged EBNA1 is similar to our previous observations with untagged EBNA1 [18]. In contrast to EBNA1, APE1/Ref-1 had no effect on transactivation by Tat (Figure 5A). APE1/Ref-1 did not augment EBNA1's ability to transactivate by increasing EBNA1 expression (Figure 5B).

### Selenium and over-expression of thioredoxin reductase 1 (TRR1) selectively reduce transactivation by Tat

Tat protein reduced *in vitro *is transactivation impaired when electroporated into cells [28]. Consistent with this observation, recent reports indicate that RNA-interference mediated depletion of increases Tat's capacity to transactivate in the monocytic cell-line U937, and Tat binds TRR1 *in vitro *[24]. TRR1 is a cytoplasmic seleno-enzyme that recycles thioredoxin by reducing disulfide bonds [53]. In addition, TRR1 also directly reduces disulfide bonds in a number of substrate proteins [24,53]. The HIV-1 LTR contains binding sites for multiple redox-sensitive transcription factors including NFkB and Sp1. The effect of TRR1 on Tat's ability to transactivate the HIV-1 LTR was performed using an LTR derivative in which the NFkB sites were deleted [24]. However this LTR-based Tat reporter still contains intact Sp1 sites, a transcription factor that is redox regulated by thioredoxin and by TRR1 [54].

**Figure 6:**
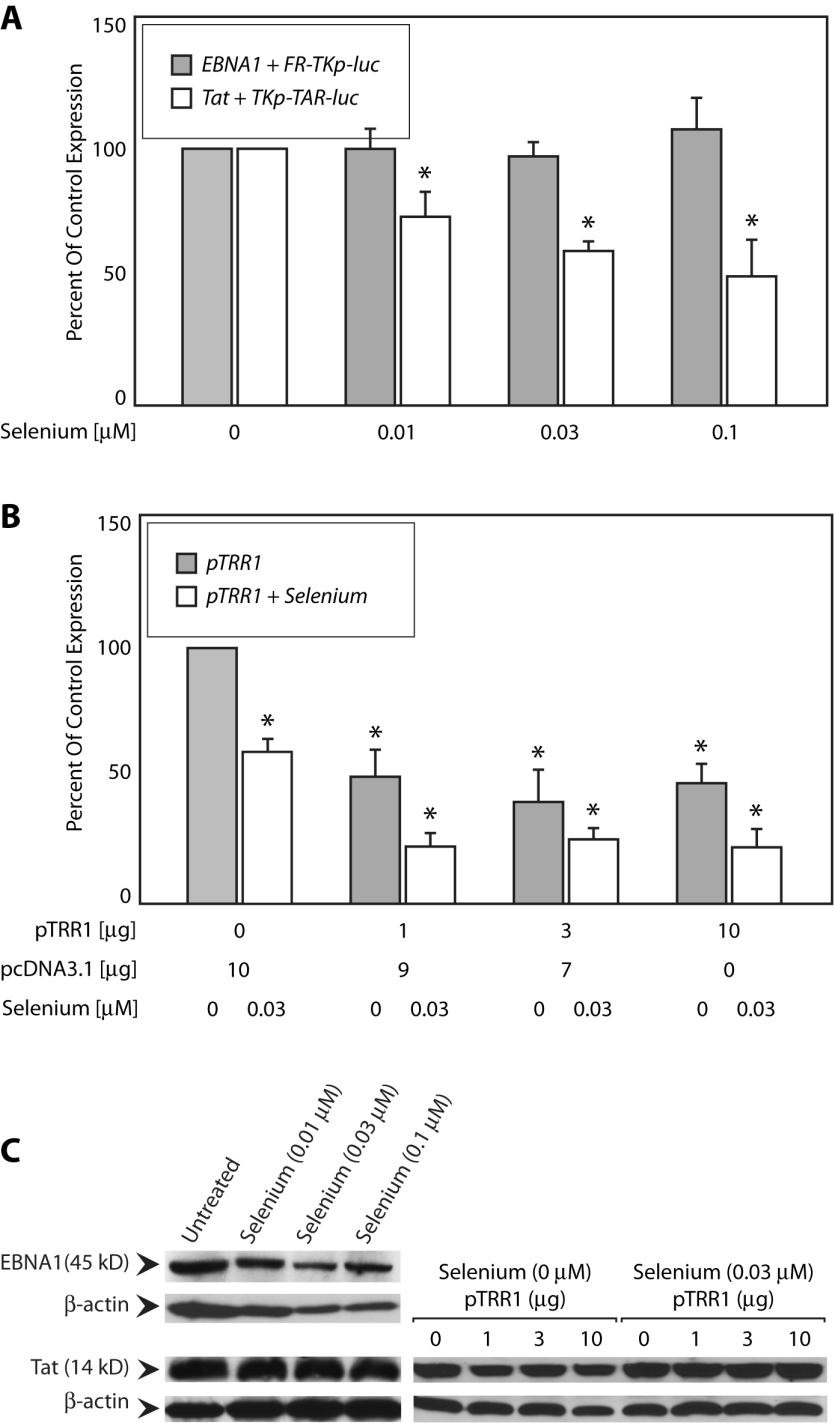
**Selenium and thioredoxin reductase 1 (TRR1) reduce transactivation by Tat**. (A) Transfected cells were split 6 hours post-transfection into aliquots and exposed to the indicated concentrations of sodium selenite for 18 hours before analysis. The inset legend indicates the columns corresponding to each effector/reporter combination. Asterisks indicate statistical significance by the Wilcoxon rank-sum test (p < 0.05) for selenium treated samples compared to vehicle treated samples (B) C33a cells were co-transfected with Tat expression and reporter plasmids and indicated amounts of a TRR1 expression plasmid. pcDNA3.1 was used to normalize the amount of DNA used per transfection. Transfections was split six hours post-transfection, and half the transfected cells were exposed to 0.03 μM sodium selenite for an additional 18 hours before analysis. Transactivation is expressed as a percent of transactivation observed in the absence of co-transfected pTRR1 or added sodium selenite (control conditions). The inset legend indicates the columns corresponding to co-transfected pTRR1 alone, or co-transfected pTRR1 with sodium selenite addition. Asterisks indicate statistical significance by the Wilcoxon rank-sum test (p < 0.05) for TRR1 transfected cells compared to controls in which pcDNA3.1 was co-transfected with reporter and effector plasmids, and cells were not exposed to sodium selenite. (C) Immunoblot analysis indicates that treatment with sodium selenite does not alter the expression of EBNA1 or Tat. In addition, co-transfected pTRR1 does not affect the expression of Tat in the presence of absence of 0.03 μM sodium selenite added to the media. β-actin was used as a loading control.

The minimal TK promoter used in the TKp-TAR-luciferase reporter described here lacks recognition sites for Sp1 or any other major redox-regulated transcription factor. Therefore, we tested whether activating TRR1 by the addition of selenium (Figure 6A), or over-expression of TRR1 (Figure 6B), would decrease Tat's ability to transactivate. For the data shown in Figure 6A, C33a cells transfected with effector and reporter plasmids were split six hours post-transfection, and aliquots exposed to increasing concentrations of selenium (0.01 - 0.1 μM). As shown in Figure 6A, the addition of 0.01 μM and higher concentration of selenium significantly decreased Tat's capacity to transactivate. At 0.1 μM, Tat transactivated TKp-TAR-luciferase at 55% the level observed in the absence of selenium. Selenium did not affect EBNA1's ability to transactivate FR-TKp-luciferase. Next, the effect of TRR1 over-expression was tested (Figure 6B). Over-expressed TRR1 negatively affected Tat's capacity to transactivate significantly, even in the absence of additional added selenium (Figure 6B), such that co-transfection of 1 μg of a TRR1 expression plasmid reduced Tat's capacity to transactivate to 45% of control. No further effect was observed with higher amounts of the co-transfected TRR1 expression plasmid. Over-expression of TRR1 had no effect on EBNA1's ability to transactivate (data not shown). While we were initially surprised that over-expression of TRR1 decreased Tat's capacity to transactivate even in the absence of added selenium, it is possible that the over-expressed TRR1 uses the pre-existing intracellular selenium pool to form the active enzyme. Alternatively, it has been reported that TRR1 reduces many disulfide bonds in the absence of selenium [53]. We also tested whether the combination of over-expressed TRR1 and selenium addition would further decrease Tat's capacity to transactivate in cells that over-express TRR1. As shown in Figure 6B; addition of 0.03 μM selenium reduced transactivation by Tat to 25% in cells co-transfected with 1 μg of the TRR1 expression plasmid. Addition of selenium had no effect on the expression of EBNA1 or Tat, and over-expression of TRR1 also had no effect on Tat expression (Figure 6C), confirming that the decrease in transactivation did not result from a decrease in Tat levels.

Because TRR1 reduces oxidized thioredoxin that acts to reduce disulfide bonds, we also tested whether over-expression of thioredoxin affected Tat's activity. In multiple experiments, thioredoxin did not affect transactivation by Tat (data not shown), thus confirming the observation that TRR1 directly interacts with Tat to affect transactivation [24].

## Discussion

Virus infection results in different outcomes for HIV-1 and EBV. Infection by HIV-1 results in the depletion of a T-cell subset, whereas EBV immortalizes naive B-cells. EBV-immortalized cells proliferate in lymph nodes, a relatively anoxic niche within the body, and EBV-positive lymphomas also proliferate at anoxic sites [19,43,44]. In peripheral circulation, EBV-immortalized cells are found as quiescent memory-B cells [44]. The effect of pO_2 _alterations is less clear for HIV-1 pathogenesis. In general, higher levels of HIV-1 RNA are detected in peripheral circulation, while lower levels are observed in anoxic niches [45,46].

It is likely that numerous physiological and cellular conditions result in differences observed for these viruses in differing physiological niches. On the basis of the results from this study, we speculate that redox-dependent function of two critical viral transactivators may underlie niche-dependent differing outcomes of infection.

EBNA1 transactivates the expression of a subset of EBV genes required to drive the proliferation of EBV-infected cells. Therefore, hypoxic/anoxic conditions that increase transactivation of these genes by EBNA1 may contribute to the proliferative phenotype displayed by EBV-infected cells in lymph nodes and other anoxic sites.

In the absence of Tat, HIV-1 mRNA and genomic transcripts are prematurely terminated. Our results, and those of others, indicate that oxidizing conditions increase the expression of a TAR-dependent reporter in the presence of Tat [24]. In addition, our results indicate that hypoxia decreases the activity of Tat, similar to other recent observations [30]. Reduction of Tat with chemical agents also decreases its transactivation capacity [28]. Together these observations contrast with earlier observations that anoxic conditions increased HIV-1 RNA expression [55]. This difference could potentially arise from the activation of cellular transactivators under hypoxic conditions or cellular differences. Superficially, our results also contrast with those reported recently on the effect of bacterially expressed, exogenously added Tat for HIV-1 infection of primary T-cells [4]. In this study, under hypoxic conditions, exogenously provided Tat primed T-cells for HIV-1 infection. The reason for this difference is unknown; it may be pertinent that we have examined the activity of Tat on a TAR-dependent reporter, but the mechanism by which exogenously added Tat primes naive T-cells for infection by HIV-1 is unknown. In this context, we note that administration of the reducing agent, N-acetyl cysteine, inhibits HIV-1 expression in a chronically infected cell model [56,57]. It is possible that this decreased expression results by reducing the capacity of Tat to transactivate.

Finally, at a molecular level, our results can be interpreted to indicate that oxidative stress modifies sulfhydryl groups on EBNA1 and Tat differently. Consistent with results reported previously [24], the effects of beta-mercaptoethanol and over-expression of TRR1 suggests that oxidized cysteines in Tat exist as disulfide bonds. In contrast, neither beta-mercaptoethanol nor TRR1 have any effect on EBNA1, suggesting the EBNA1 oxidation does not result in disulfides. This conclusion is supported by the observation that APE1/Ref-1, which reduces sulfenic acid to sulfhydryl, augments transactivation by EBNA1.

In summary, our studies have unexpectedly revealed dramatically different effects of oxidative stress on these two viral transactivators. This difference may reflect the physiological sites that cells infected by EBV and HIV-1 traffic to. The differential effect of oxidative stress has implications for potential therapeutic interventions that target oxidative stress in patients co-infected with both viruses.

## Conclusions

The activity of EBNA1, a critical EBV transactivator, and Tat, a critical HIV-1 transactivator, are modulated by redox. Oxygen tension and oxidative stress have strikingly opposite effects on the capacity of these proteins to transactivate. Hypoxia increases transactivation by EBNA1, while decreasing Tat transactivation. Conversely, reactive oxygen species generated by menadione and paraquot reduce transactivation by EBNA1 but increase Tat function. The cellular redox modulators APE1/Ref-1 and TRR1 have transactivator-specific effects. APE1/Ref-1 augments EBNA1's capacity to transactivate with no effect on Tat. On the other hand, TRR1 reduces Tat's capacity to transactivate without affecting EBNA1. This data permits us to propose that the redox-dependent functions of EBNA1 and Tat may underlie the behavior of EBV and HIV infected cells within physiological niches that differ in oxygen tension.

## Competing interests

The authors declare that they have no competing interests.

## Authors' contributions

ATW was responsible for experimental design, conducting experiments and writing the manuscript. GS was responsible for conducting experiments. AA was responsible for conducting experiments and writing the manuscript. All three authors have read and approved the final manuscript.

## Supplementary Material

Additional file 1 **Zinc depletion, hypoxia, and oxidative stress do not affect basal transcription from the minimal TK promoter**. (A) C33A cells transfected with TKp-luciferase (AGP47) were split 6 hours post-transfection such that one aliquot was exposed to 1 μM of TPEN for 18 hours prior to being assayed, (B) Cells transfected as in A were split 6 hours post-transfection and exposed to an additional 18 hours to normoxia (N) or hypoxia (H), (C) Cells transfected and split as in A were exposed to the indicated concentrations of paraquot, and (D) β-mercaptoethanol. Transactivation is expressed as a percent of expression under control conditions. Chelation of zinc, oxygen tension and oxidative stress did not significantly alter expression from the minimal HSV-1 promoter. Click here for file
