# Supplementary figures and images for: Diametrically opposed effects of hypoxia and oxidative stress on two viral transactivators
Source: Virol J. 2010 May 10;7:93. doi: 10.1186/1743-422X-7-93 (PMC2874542; doi:10.1186/1743-422X-7-93)

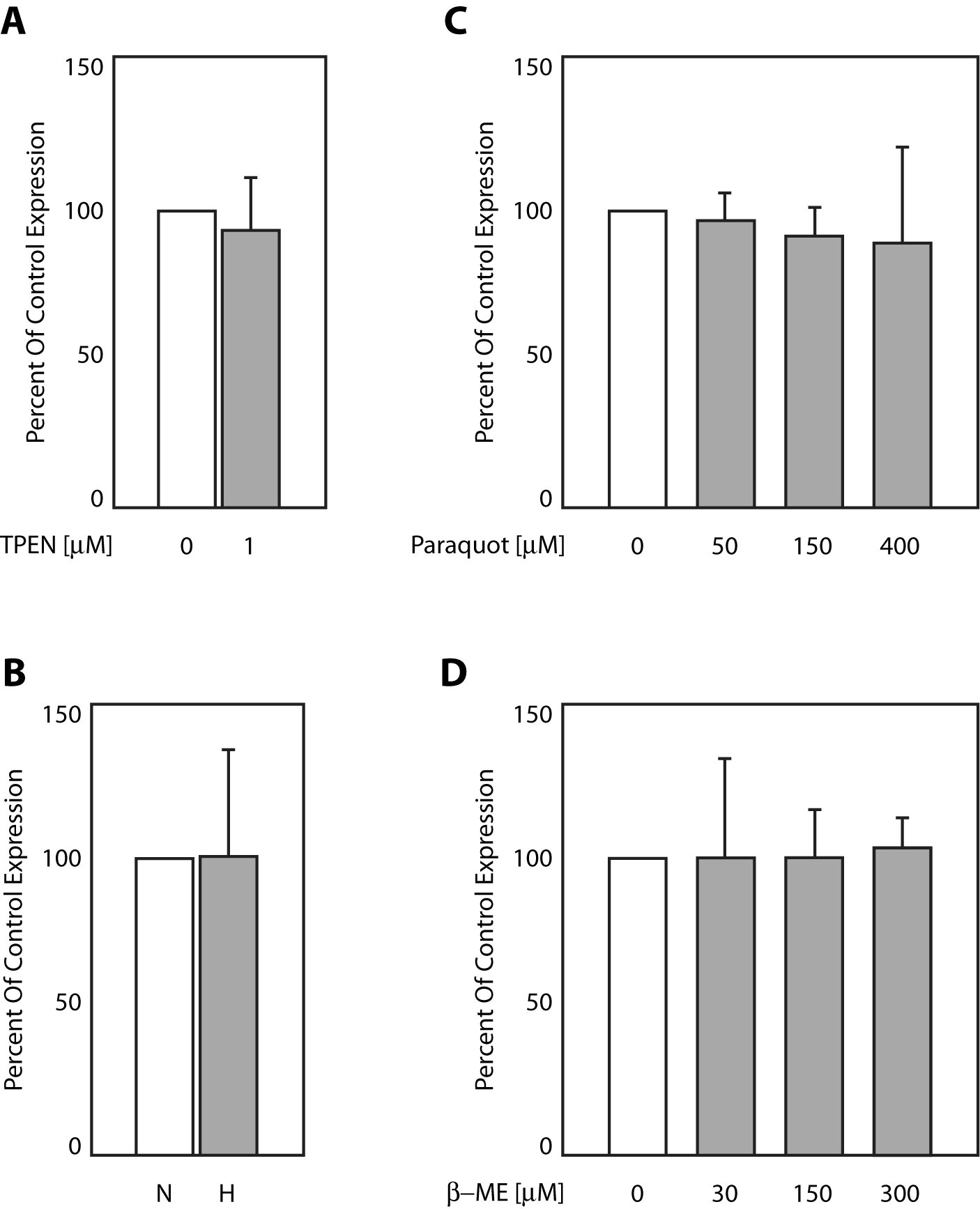

Supplement: Additional file 1 — Zinc depletion, hypoxia, and oxidative stress do not affect basal transcription from the minimal TK promoter. (A) C33A cells transfected with TKp-luciferase (AGP47) were split 6 hours post-transfection such that one aliquot was exposed to 1 μM of TPEN for 18 hours prior to being assayed, (B) Cells transfected as in A were split 6 hours post-transfection and exposed to an additional 18 hours to normoxia (N) or hypoxia (H), (C) Cells transfected and split as in A were exposed to the indicated concentrations of paraquot, and (D) β-mercaptoethanol. Transactivation is expressed as a percent of expression under control conditions. Chelation of zinc, oxygen tension and oxidative stress did not significantly alter expression from the minimal HSV-1 promoter. [file 1743-422X-7-93-S1.TIFF]
